# An Acute Leukaemia Masquerading as Immune Thrombocytopaenic Purpura (ITP)? A Case Report

**DOI:** 10.4137/ccrep.s2394

**Published:** 2009-04-17

**Authors:** J.A. Olaniyi

**Affiliations:** Department of Haematology, University College Hospital, Ibadan, Nigeria.

**Keywords:** acute myeloid leukaemia, immune thrombocytopaenic purpura, pregnancy, bleeding, M4 subtype

## Abstract

This is a case report of a 35 year old female with diagnosed Immune Thrombocytopaenic Purpura (ITP) that was strangely followed by acute myeloid leukaemia at 10 months post diagnosis of ITP. She was managed as ITP using prednisolone 45 mg daily for 10 months with good response. She also synchronously carried a pregnancy to term and safe delivery. Shortly after delivery, she represented with gingival bleeding and peripheral film review and subsequent bone marrow cytology was in keeping with AML-M4 subtype. She died shortly after diagnosis without being able to receive chemotherapy.

## Introduction

Many aetiological factors predisposing to Acute Myeloid Leukaemia (AML) are well established in literature.^1^–^4^ However, as far back as 1978; Ihara et al.^5^ reported a case of AML in a 29 year old female in 10 years course of ITP with episodes of splenectomy and of safe delivery of a baby girl. Also reported recently is AML that strangely emerged from a diagnosed breast carcinoma patient within one month of diagnosis.^6^ Extensive literature search failed to reveal any link in respect of pathogenesis between AML and ITP. While acute leukaemia is known to be an aggressive disease that usually presents with features of bone marrow failure, presence of excess of blasts in the bone marrow, spillage of blasts into the peripheral circulation and evidence of multiple tissue infiltration;^1^,^7^ ITP is known to be associated with an abnormal autoantibody, usually immunoglobulin G (IgG) with specificity for GPIIb/IIIa and GPIb/IX or more platelet membrane glycoproteins; which bind to circulating platelet membrane.^8^–^10^ Auto-antibody coated platelets induce Fc receptor mediated phagocytosis by mononuclear macrophages primarily in the spleen.^9^ The resultant thrombocytopaenia manifests as bleeding tendency, easy bruising (purpura), or extravasations of blood from capillaries into skin and mucous membrane (petechiae). ITP and AML therefore have in common, thrombocytopaenia and its sequelae.

ITP is known to be a diagnosis of exclusion and acute leukaemia happens to be a strong differential diagnosis. Other causes of thrombocytopaenia include myelophthisic marrow infiltration, myelodysplasia, aplastic anaemia, adverse drug reaction, systemic lupus erythematosus, HIV and other viral infections etc. All these must be excluded in order to settle for a diagnosis of ITP.

In this index case, evolving acute leukaemia was strongly considered as a differential diagnosis from day one but there was no strong evidence either in the marrow or peripheral film to support a diagnosis of acute leukaemia until 11 months after when a diagnosis of AML-M4 (myelomonocytic) subtype was made. This is an unusual presentation of acute leukaemia and hence the need for this case presentation.

## Case Presentation

A 34-year-old lady (Hosp No 918233), presented 19th January 2001 with a day history of nose-bleed, gum bleed and appearance of dark-red spots on the skin. The bleeding was unprovoked. Further questioning revealed that she had, in the past 4 years, been noticing gingival bleeding after brushing the mouth and had noticed dark blue/red patches on her limbs. She had also experienced heavy menstrual flow for five consecutive months in the preceding year which required Gynecologist expert service in controlling the bleeding. She had used Ibuprofen for a while for pain control. There was no associated fever, weight loss or excessive night sweat. She was the 6th of 7 siblings of 4 males and 3 females. No similar history in the female siblings.

Examination revealed mild pallor; tiny cervical and axillary’s lymphadenopaty and also presence of multiple petechiae and ecchymotic patches on both forearms and lower limbs. Examination of each of the systems was essentially normal except for a tipped liver below the coastal margin. An impression of chronic ITP was made.

The initial full blood count (FBC) showed a packed cell volume (PCV) of 27%, white cell count {WBC} of 4,700/mm^3^ with a differential revealing essentially relative lymphocytosis. Direct combs test was negative and human immunodeficiency virus (HIV) 1 and 2 were none reactive. The coagulation profile was not deranged. Chest X-Ray and Abdominal Ultrasound at presentation were within normal limits. L: E preparation {Lupus Erythematosus} was positive.

Bone marrow Aspiration examination showed essentially increased megakaryocytes with budding and normal erythropoiesis and myelopoiesis. Reactive lymphoid cells were seen.

Treatment for ITP was commenced with intravenous hydrocortisone stat, then oral prednisolone at 45 mg daily in three divided doses. We had to give 2 units of fresh whole blood (when platelet concentrate was in short supply) in view of life threatening anaemia and thrombocytopaenic bleeding. After two weeks of steroid therapy, platelet count rose to 95,000/mm^3^ and all bleedings tendencies subsided. She was discharged on steroid medication.

After about 13 weeks of therapy, patient informed that she had missed her period. She presented then with a gum bleed while temporarily off steroid. Her FBC then showed a PCV of 29%, WBC of 4.8 × 10^9^/L and a platelet count of 65,000/mm^3^. The Obstetrician was consulted at this point and thereafter co-managed. Within a week of steroid recommencement, the platelet count was 113,000/mm^3^ and the PCV was 30.4%. The Obstetrician put her gestational age at 15 ± 2 weeks and established that she had voluntarily terminated six pregnancies. She was actually Gravida 8, Para 2, with 6 abortions and 2 alive. First confinement was in 1982.

The suspicion of acute leukaemia began in November 6, 2001 when she presented again with gingival bleeding and multiple fresh petechiae on the skin in spite of steroid therapy. FBC then showed a PCV of 36%, a WBC of 7000/mm^3^ and a platelet count of 15,000/mm^3^. The review of blood film showed the presence of fairly large primitive admixture of precursor granulocytes and monocytoid cells, there nuclei having open chromatin with 2–4 nucleoli, low nuclear cytoplasmic ratio and the monocytoid cells have pale blue agranular cytoplasm. Repeat bone marrow aspiration showed features consistent with AML-M4.

She was resuscitated and written up for combination chemotherapy with Daunorubicin, Cytosine arabinoside and Etoposide or 6-Thioguanine (DAE or DAT) but declined treatment because of financial constraint.

She opted to go to her village and a referral was written to the nearest Teaching Hospital. She did not present to any hospital and she died two weeks thereafter.

## Discussion

It appears that the index patient fulfilled the diagnostic criteria of ITP and responded satisfactorily to steroid therapy. Although the patient was in and out of hospital admission; she was able to carry a pregnancy to term with safe spontaneous virginal delivery of a live baby boy. Patient presented with persistent thrombocytopaenia, multiple thrombocytopaenic bleeding, evidence of increased megakaryocytes in the marrow and absence of morphologically abnormal cells, particularly blasts and dysplastic cells in the marrow. Diagnosis of AML-M4 was made 11 months after initial diagnosis of ITP. This appears to be an unusual presentation.

Experience in clinical practice had shown that there is little uniformity in the clinical presentation of acute leukaemia; while some patients are remarkably asymptomatic, others are seriously ill.^1^ However, review of literature is yet to show acute leukaemia in evolution through ITP.

In frank acute leukaemia, abnormal malignant blast cells derived from genetic alteration within a single stem cell accumulate; displace/replace normal haemopoietic precursor cells of the bone marrow resulting in bone marrow failure.^1^ In this our index patient apart from persistent thrombocytopaenia with anaemia resulting from thrombocytopaenic bleeding; white cells morphology and counts remain consistently normal except for atypical lymphocytes noticed at diagnosis (refer Table 1). There was no evidence of blast accumulation in the marrow/spillage into circulation until after 11 months post diagnosis of ITP.

It is known that AML could emerge from myelodysplastic syndrome (MDS) and other haematological disorders like the non-leukaemic Myeloproliferative disorders (MPDs), Aplastic anaemia, Paroxysmal nocturnal Haemoglobinuria (PNH) but not ITP.^1^

Evolution of acute leukaemia is considered to be a multistep process with mechanisms of leukaemic transformation arising from inherited aetiological factors like Downs syndrome, kline-felter’s syndrome, Bloom’s syndrome, Fanconi’s Anaemia, ataxia telangectasia etc. Patients with Fanconi anaemia in particular have elevated risks of developing AML,^3^,^4^ Acute myelomonocytic leukaemia (AMML) which happens to be the subtype the patient developed and Monocytic (M5) subtypes are the most commonly observed.^4^

Other factors that initiate such multistep process include chemicals like benzene and alkylating agents; radioactive substances like atomic nuclear x-ray, electrical field, radioactive irradiation, Other aetiological factors include Viruses like HTLV-1, EBV and HIV also serve as indirect aetiological agents.

While thrombocytopaenia is a cardinal feature of acute leukaemia; its preceding acute leukaemia for a period of up to 11 months with outright absence of blasts in the marrow and peripheral circulation is quite unusual and has not been reported.

In retrospect, the peak of the illness of this patient coincided with early first trimester of pregnancy since her first day of presentation was 19/01/2001 and her LMP was 27/1/2001. This brings to consideration the role of pregnancy in provoking ITP or in ameliorating acute leukaemia. This becomes important in that the full picture of acute leukaemia only became apparent after delivery.

Therefore the question remains—is this chronic ITP terminating as acute leukaemia? or acute leukaemia masquerading as ITP? Junya Kuroda et al.^11^ in a retrospective study of 25 and 22 with initial diagnosis of ITP and MDS respectively; equally discovered that 5 of the 25 diagnosed ITP patients were found to have inexplicable refractory anaemia and or neutropaenia which progressed slowly but did not transform to leukaemia. This case report has raised more questions than answers but the possible underlying problem worthy of consideration in this patient is haematologic abnormalities arising from Fanconis anaemia^4^ and other inherited disorders predisposing to acute myeloid leukaemia listed earlier in this discussion. Unfortunately intense molecular study was not carried out on this patient not only for lack of standard laboratory and manpower but also because the patient did not give us room to explore the avenues.

This case report stresses the need for close monitoring of ITP cases and enhanced diagnostic procedures in refractory ITP to uncover secondary reasons for thrombocytopaenia. Intense molecular study is required to find out background genetic abnormalities underlying a clinical case so as to be able to predict the evolutionary trend.

## Figures and Tables

**Table 1:** Shows the serial FBC and differentials of white cells of index patient.

DATE	PCV	WBC × 10^9^/L	PLAT × 10^9^/L	LMPH × 10^9^/L	MONO × 10^9^/L	GRANU × 10^9^/L
19/2/01	27	4.7	35	42	9	50
28/2/01	32	10.9	52	28	05	67
6/3/01	33	15.4	95	26	07	67
4/4/01	30.2	3.8	175	52.7	6.7	40.6
5/4/01	30	3.8	–	–	–	–
26/4/01	29	4.8	64	–	–	–
4/5/01	30	–	113	32.8	10	57
19/7/01	31.5	11.4	187	29.9	9.1	66.0
23/8/01	24	6.0	136	33.5	6.5	60.0
8/11/01	34	5.4	–	–	–	–
15/11/01	33	7′3	155	41	8	50.9
6/12/01	37	5.6	71	17	04	43
10/12/01	34	4.0	17	29	11	60

**Abbreviations:** PCV, Packed cell volume; Mono, monocyte percentage; WBC, Total white cell count; Granu, Granulocyte percentage; Plat, platelet count; Lymph, lymphocyte percentage.
